# Elite Haplotypes of a Protein Kinase Gene *TaSnRK2.3* Associated with Important Agronomic Traits in Common Wheat

**DOI:** 10.3389/fpls.2017.00368

**Published:** 2017-03-28

**Authors:** Lili Miao, Xinguo Mao, Jingyi Wang, Zicheng Liu, Bin Zhang, Weiyu Li, Xiaoping Chang, Matthew Reynolds, Zhenhua Wang, Ruilian Jing

**Affiliations:** ^1^College of Agronomy, Northeast Agricultural UniversityHarbin, China; ^2^National Key Facility for Crop Gene Resources and Genetic Improvement, Institute of Crop Science, Chinese Academy of Agricultural SciencesBeijing, China; ^3^College of Life Science and Technology, Gansu Agricultural UniversityLanzhou, China; ^4^International Maize and Wheat Improvement CenterTexcoco, Mexico

**Keywords:** *TaSnRK2.3*, association analysis, haplotype, functional marker, stem water-soluble carbohydrates

## Abstract

Plant-specific protein kinase SnRK2s play crucial roles in response to various environmental stimuli. *TaSnRK2.3*, a SnRK2 member, was involved in the response to multiple abiotic stresses in wheat. To facilitate the use of *TaSnRK2.3* in wheat breeding, the three genomic sequences of *TaSnRK2.3*, originating from the A, B, and D genomes of hexaploid wheat, were obtained. Sequence polymorphism assays showing 4 and 10 variations were detected at *TaSnRK2.3-1A* and at *TaSnRK2.3-1B*, respectively, yet no variation was identified at *TaSnRK2.3-1D*. Three haplotypes for A genome, and two main haplotypes for B genome of *TaSnRK2.3* were identified in 32 genotypes. Functional markers (2.3AM1, 2.3AM2, 2.3BM1, 2.3BM2) were successfully developed to distinguish different haplotypes. Association analysis was performed with the general linear model in TASSEL 2.1. The results showed that both *TaSnRK2.3-1A* and *TaSnRK2.3-1B* were significantly associated with plant height (PH), length of peduncle and penultimate node, as well as 1,000-grain weight (TGW) under different environments. Additionally, *TaSnRK2.3-1B* was significantly associated with stem water-soluble carbohydrates at flowering and mid-grain filling stages. *Hap*-1A-1 had higher TGW and lower PH; *Hap*-1B-1 had higher TGW and stem water-soluble carbohydrates, as well as lower PH, thus the two haplotypes were considered as elite haplotypes. Geographic distribution and allelic frequencies indicated that the two preferred haplotypes *Hap*-1A-1 and *Hap*-1B-1 were positively selected in the process of Chinese wheat breeding. These results could be valuable for genetic improvement and germplasm enhancement using molecular marker assisted selection in wheat breeding.

## Introduction

During the processes of growth and development, plants are vulnerable to various kinds of abiotic stresses, including drought, high salinity, and extreme temperature because of their immobility. To survive, plants have evolved a number of ways to cope with versatile environmental stresses. Protein kinases and phosphatases, are major components of intracellular signal transduction, and play important roles in multi-environmental stress responses (Hong et al., 1997).

Diverse stress-inducible protein kinase families mainly include mitogen-activated protein kinase (Wrzaczek and Hirt, 2001), calcium-dependent protein kinase (Ludwig et al., 2004), and sucrose non-fermenting 1 (SNF1)-related protein kinase (SnRK). Most of them are activated by abscisic acid (ABA) or environmental stimuli. SNF1 protein kinase in yeast, AMP-activated protein kinase in mammals, and plant SnRK (especially SnRK1) protein are highly conserved and play pivotal roles in growth and metabolic response to cellular stresses as energy sensors.

The SnRK family is classified into three subfamilies in plants, i.e., SnRK1, SnRK2, and SnRK3, based on sequence similarity, gene structures, and expression patterns. SnRK2 is a relatively small plant-specific subfamily, encoding serine/threonine kinases. The SnRK2s contain two typical domains, viz., an N-terminal catalytic domain which plays an important role in kinase activation, and a regulatory C-terminal region involved in protein-protein interactions and possibly in ABA signaling (Huang et al., 1996; Vlad et al., 2009). The SnRK2s were further divided into three subclasses in phylogeny according to their varied activation patterns in response to ABA (Kobayashi et al., 2004). Among them, subclass III was strongly induced by ABA, weakly for subclass II, and no activation for subclass I. Accumulated evidence indicated that SnRK2 is a merging point of ABA-dependent and ABA-independent pathways in abiotic stress responses and developmental processes in plants (Fujii et al., 2011; Kulik et al., 2011). Our target gene *TaSnRK2.3* belongs to subclass II in common wheat (*Triticum aestivum* L.) (Zhang et al., 2016). In previous research, we cloned and characterized *TaSnRK2.3* in wheat, its hetero-expression resulted in improved tolerances to multiple abiotic stresses (Tian et al., 2013).

Wheat is one of the most important cereal crops worldwide, while its growth and development is severely influenced by abiotic stresses, resulting in significant reduction in grain yield. In the scenario of climate change, mining and utilization of key genes conferring tolerances to abiotic stress is regarded as an effective way to ensure a high and stable yield in wheat. However, common wheat is a hexaploid species (AABBDD) with a very large and complex genome (17.9 × 10^9^ bp), enriched in abundant repeat sequences (about 86%) (Varshney et al., 2006), hence it is still a serious challenge to directly isolate a gene and further decipher its function at the molecular level, although three genome drafts of diploid and hexaploid wheat have been constructed (Jia et al., 2013; Ling et al., 2013; International Wheat Genome Sequencing Consortium [IWGSC], 2014). Marker assistant selection (MAS) based on elite allele pyramiding is considered a potential approach to wheat improvement for complex traits. As the third generation molecular marker, single nucleotide polymorphism (SNP) featured with high abundance and stability, cost efficiency, and high-throughput scoring, has been widely used in plant heredity and breeding (Collard and Mackill, 2008; Wang et al., 2015). With the development of high density SNPs and other molecular markers, association analysis has become an efficient tool to identify the relationship between markers or polymorphism sites of target genes and traits, and has been successfully used in *Arabidopsis thaliana* (Nemri et al., 2010), rice (Agrama et al., 2007), maize (Thornsberry et al., 2001; Li et al., 2010), and wheat (Zhang et al., 2015; Li et al., 2016). Mining causative molecular polymorphisms and developing functional markers are fundamental to stacking superior alleles of key genes in genetic improvement of crops using MAS methods (Wang et al., 2016).

To facilitate utilization of *TaSnRK2.3* in wheat molecular breeding by MAS, our research mainly concentrated on: (i) isolating and characterizing three genomic sequences of *TaSnRK2.3* in common wheat, (ii) identifying polymorphism sites and developing functional markers in *TaSnRK2.3-1A/1B*, (iii) identifying favorable allelic variations and haplotypes for *TaSnRK2.3-1A/1B* by association analysis, (iv) revealing the distribution of preferred genotypes in varieties released in different years and geographical environments in China. The results can offer valuable information for wheat improvement.

## Materials and Methods

### Plant Materials and Measurement of Agronomic Traits and Stem Water-Soluble Carbohydrates

Common wheat cultivar Hanxuan 10 with remarkable tolerance to drought stress was used for genomic sequence isolation of *TaSnRK2.3* and gene structure analysis. Twelve accessions of various wheat species, including three A genome accessions (*Triticum urartu*) (UR204, UR206, and UR207), three S genome accessions (*Aegilops speltoides*, the putative B genome donor) (Y2003, Y2033, and Y2017), three D genome accessions (*Ae. tauschii*) (Y125, Y225, and AE38), and three AB genome accessions (*T. dicoccoide*) (DS1, PS5 and PS9) were selected for target fragment isolation and genomic origin identification.

Thirty-two accessions/genotypes with wide variation screened by SSR markers, were initially chosen to re-sequence for polymorphism analysis. Three hexaploid wheat germplasm populations were selected for different research purposes. Population 1 (262 accessions/genotypes) was firstly employed for association analysis. The accessions were mainly released in the Northern winter wheat and Yellow and Huai River valley facultative wheat zones (Zhang et al., 2015). Population 2 (157 landraces/genotypes) and Population 3 (348 modern cultivars/genotypes) were used to determine temporal haplotypes and analyze geographic distribution aiming to functionally validate *TaSnRK2.3-1A/1B* markers. Population 2 was mainly from the Chinese wheat mini-core collection representing more than 70% of the genetic diversity of the total Chinese germplasm collection; Population 3 came from the Chinese wheat core collection (Hao et al., 2008; Hao et al., 2011). The two populations (2 and 3) including genotypes from all the 10 Chinese wheat production zones were selected from 23, 705 accessions released or collected in China (Zhang et al., 2002; Dong et al., 2003; Hao et al., 2008).

Population 1 was planted in 10 environments (year × site × water regime combinations) including Changping (116°13′E, 40°13′N), Beijing, in 2010 and 2012, and Shunyi (116°56′E, 40°23′N), Beijing, in 2010, 2011, and 2012. The field experiments were grown under well-watered (WW) and drought-stressed (DS) regimes. The WW plots were irrigated with 750 m^3^ ha^-1^ (75 mm) at each of pre-overwintering, booting, flowering, and grain filling stages (total 300 mm applied as irrigation), while DS plots were rain-fed. The rainfall during the growing seasons were 131 mm in 2010, 180 mm in 2011, and 158 mm in 2012. Measured agronomic traits included 1,000-grain weight (TGW), plant height (PH), peduncle length (PLE), length of penultimate node (LPN), spike length, number of spikes per plant, total number of spikelets per spike, number of sterile spikelets per spike, and grain number per spike.

Stem water-soluble carbohydrates (SWSC) are an important carbon source for grain filling in wheat. They are mainly composed of fructans, sucrose, glucose, and fructose, with the main reserve as fructans at the late stage of WSC accumulation (Ruuska et al., 2006). We obtained SWSC data of Population 1 under WW and DS conditions. SWSC were measured by near-infrared reflectance spectroscopy (MAP multi-purpose FT-NIR analyzer) as previously described (Wang et al., 2011). Five main stems were cut 1 cm above the soil surface at the flowering, mid-grain filling (14 days after flowering), and maturity stages. Leaf blades were removed from samples, and stem samples were cut into two parts, namely, peduncle, and the lower internodes except for peduncle. The WSC was determined for peduncle, lower internode and total stem, using different near-infrared reflectance spectroscopy (NIRS) regression models, which were developed for quantitative determination of WSC using modeling samples of 150 DH (Hanxuan 10 × Lumai 14) lines (Wang et al., 2011).

### Isolation and Sequencing of Genomic Region Surrounding *TaSnRK2.3*

For each accession, genomic DNA was extracted from young leaves by cetyltrimethylammonium bromide (CTAB) method (Stewart and Via, 1993). Based on known cDNA information, a primer pair GF/R (**Table 1**) was obtained to amplify the genome sequence of *TaSnRK2.3*. TransStart Fast *Pfu* DNA polymerase was used in PCR amplification (TransGen, Inc). PCR products were extracted and cloned into *pEASY*-Blunt vector, 24 clones for each sample were randomly selected for sequencing by DNA Analyzer 3730XL. To get the whole sequence of *TaSnRK2.3*, both M13 and three overlapping primers (**Table 1**) were used for sequence walking. So the sequence of each clone was obtained by assembling five overlapping sequences with the SeqMan program in DNAStar. The genomic origin of each of the sequences were confirmed by comparing them with that from diploid and tetraploid species based on clustalW analysis with the MegAlign program.

**Table 1:** Primers used for genomic fragment isolation, sequencing and marker development.

Primer set	Nucleotide sequence (5′ to 3′)	Experimental purpose
GF	GTTTGCTCTGAGTTGGTGCTTC	
GR	CAGTAATAATGTACACAGCGATAG	A, B, and D genomic fragment amplification
M13F	TGTAAAACGACGGCCAGT	
M13R	CAGGAAACAGCTATGACC	
SeqF1	ACTCGTTAGTCGTTTGATAAGTT	
SeqF2	GGGCGATTCAGTGAAGAT	
SeqR1	CATGTGACATCTCAGAACCAT	Sequencing primers for *TaSnRK2.3*
AGF	TTCACAGTCGGTTTCGTTCG	A genome specific target fragment amplification
BGF	TAAGTACCGCTTATGACAATCTGTGGTT	B genome specific target fragment amplification
AG-*Hha*I-F	TACAACATAGAACTTTAGTAATGGACAGCG	
AG-*Hha*I-R	TCACGCCGCAGGACCAA	Marker 2.3AM1 developed for SNP-1898 (C/T)
AG-*Xba*I-F	CTTCAAGGAGCCCGAGACG	
AG-*Xba*I-R	TTCTTGTTTCAGCAAACCGTACTC	Marker 2.3AM2 developed for SNP-2905 (A/G)
BG-*N1a*III-F	AACTCTGATCTGAACACGAATGCA	
BG-*N1a*III-R	GTGATCCTCTTCAGAGTTTCAGACAC	Marker 2.3BM1 development for SNP-2153 (C/T)
BG-*Ava*II-F	AAGGCAGAAAACTTTGAATCATAAC	
BG-*Ava*II-R	TCCTCATCGCTCTGCAGCTCGGCCAGGTC	Marker 2.3BM2 development for SNP-2638 (C/G)

### Functional Marker Development

A total of four functional markers (2.3AM1, 2.3AM2, 2.3BM1, 2.3BM2) were developed based on four selected polymorphism sites, in order of 1898 bp (C/T) and 2905 bp (A/G) of A genome; 2153 bp (C/T) and 2638 bp (C/G) of B genome. These markers were designed with a specific mismatch in the primer to introduce a restriction enzyme recognition site using an available program dCAPS Finder 2.0^1^. Basically, the four systems of PCR and digestion were similar. Genotyping was performed by two rounds of PCR. Firstly, genome specific primer pairs were used to amplify fragments from chromosome 1A/1B in all accessions. The second round of PCR was performed as follows: the first round of PCR product was diluted 50 times, then taking 1 μl as template for the second round of PCR. The annealing temperatures and extension time were set depending on primer pairs and expected PCR product lengths. The PCR products were resolved by electrophoresis in 4% agarose gel after digestion with corresponding restriction enzymes. The primers were listed in **Table 1**.

### Association Analysis

TASSEL 2.1 was used to identify significant associations between haplotypes and agronomic traits for Population 1. The general linear model (GLM) was performed using population structure Q matrix, which listed the estimated membership coefficients for each individual in each cluster. Associations were considered significant at *P* < 0.05. Different effects of haplotype on traits were analyzed by one-way ANOVA using SPSS 16.0 software, and followed by the least significant difference (LSD) method at *P* < 0.05 (even 0.01).

## Results

### Genetic Characterization of *TaSnRK2.3*

**FIGURE 1:**
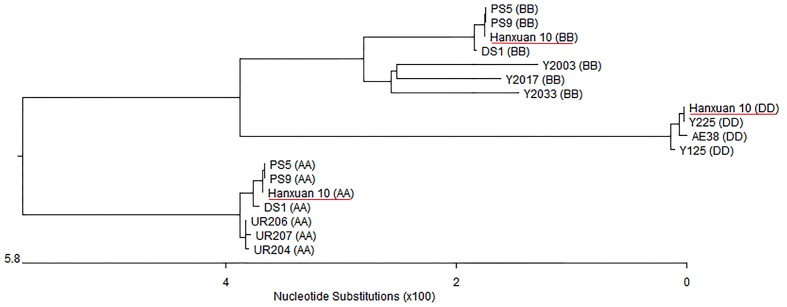
**Genomic origin identification of *TaSnRK2.3s* based on phylogenetic tree constructed with MegAlign program**. AA, *Triticum urartu*; SS, *Aegilops. speltoides* (closely related to BB); DD, *Ae. Tauschii*; AABB, *T. dicoccoide*; AABBDD, hexaploid accession Hanxuan 10.

Based on *TaSnRK2.3* cDNA, three genomic sequences of *TaSnRK2.3* were isolated from Hanxuan 10 using the primer pair GF/R flanking the open reading frame. The sequencing results indicated that the three types of sequences obtained were consistent with the previous results (Tian et al., 2013). By comparing different *TaSnRK2.3* genomic sequences among the ploidy levels of different wheat species, the genomic origin was identified (**Figure 1**). Since *TaSnRK2.3* was located on chromosome 1A, 1B, and 1D (Tian et al., 2013), we named the three copies *TaSnRK2.3-1A*, *TaSnRK2.3-1B*, and *TaSnRK2.3-1D*, respectively.

**FIGURE 2:**
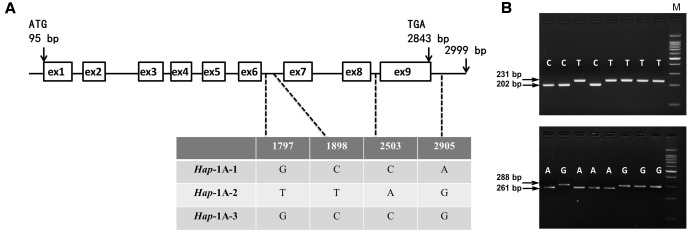
**Nucleotide polymorphisms and functional marker development of *TaSnRK2.3-1A*. (A)** Four single nucleotide polymorphisms (SNPs) were detected in the non-exon regions of *TaSnRK2.3-1A*. **(B)** A dCAPS marker 2.3AM1 was developed based on SNP-1898 (C/T) in the upper segment. Digestion of the amplified 231 bp fragment with *Hha*I produced fragments of 202 bp/29 bp for accessions with SNP-1898C, yet unacted for accessions with SNP-1898T. Similarly, a CAPS marker 2.3AM2 from SNP-2905 (A/G) was also developed in the lower segment. M, 100 bp DNA Ladder.

**FIGURE 3:**
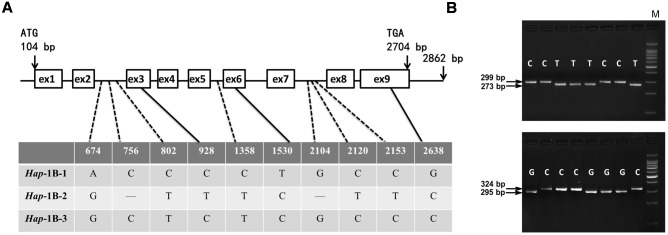
**Nucleotide polymorphisms and functional marker development of *TaSnRK2.3-1B*. (A)** Eight SNPs and two InDels were detected in the coding region of *TaSnRK2.3-1B*. “—” indicates deletion. **(B)** CAPS marker 2.3BM1 was developed based on SNP-2153(C/T). Digestion of the amplified 299 bp fragment with *N1a*III produced fragments of 273 bp/26 bp for accessions with SNP-2153T, and a single 299-bp band for accessions with SNP-2153C. Similarly, a dCAPS marker 2.3BM2 for SNP-2638(C/G) was developed. M, 100 bp DNA Ladder.

Multialignment (ClustalW) assays showed that *TaSnRK2.3* consisted of nine exons and eight introns (**Figures 2**, **3**). The fragment sizes of *TaSnRK2.3-1A*, *TaSnRK2.3-1B*, and *TaSnRK2.3-1D* were 2,999, 2,862, and 2,817 bp, and their homologies ranged from 92.5 to 94.4%.

### Sequence Polymorphism Assays and Marker Development

As shown in **Figure 2**, for *TaSnRK2.3-1A*, polymorphic sites were only identified in non-exon regions in 32 accessions, and the four SNPs formed three haplotypes (*Hap*-1A-1, *Hap*-1A-2, and *Hap*-1A-3). Ten nucleotide variations (eight SNPs and two 1-bp InDels) were detected in the target fragment of *TaSnRK2.3-1B* in the 32 common wheat accessions (**Figure 3**), only SNP at 928 bp (C/T) led to an amino acid change (CCG→Pro, TCG→Ser). The 10 SNPs formed three haplotypes, i.e., *Hap*-1B-1, *Hap*-1B-2, and *Hap*-1B-3.

To distinguish the haplotypes of *TaSnRK2.3-1A*, two functional markers (2.3AM1 and 2.3AM2) were developed based on the chosen SNP sites. One was a *Hha*I cutting site at SNP-1898C, un-cut at SNP-1898T; the other was an *Xba*I cutting site at SNP-2905A, un-cut at SNP-2905G (**Figure 2**). In the same way, markers 2.3BM1 and 2.3BM2 for *TaSnRK2.3-1B* were successfully developed with restriction enzymes *N1a*III and *Ava*II (**Figure 3**).

### Association Analysis of *TaSnRK2.3-1A* Haplotypes and Agronomic Traits

**FIGURE 4:**
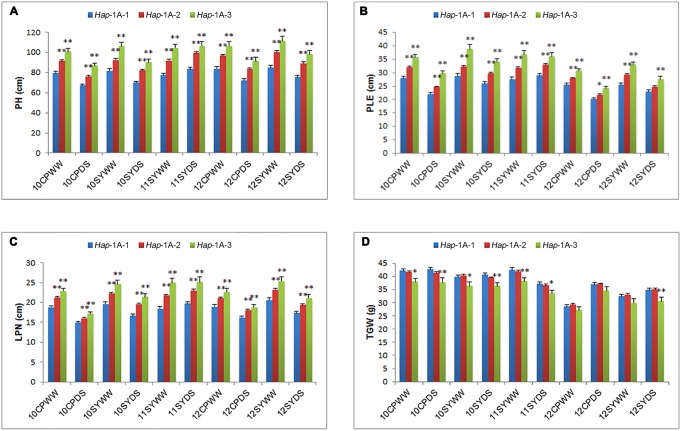
**Phenotypic comparisons of three *TaSnRK2.3-1A* haplotypes in 10 environments**. Traits include PH **(A)**, PLE **(B)**, LPN **(C)**, and TGW **(D)**. ^∗,^^∗∗^ indicate significance at *P* = 0.05 and 0.01, respectively. Error bars denote SE. See footnote to **Table 2** for abbreviations.

For *TaSnRK2.3-1A*, *Hap*-1A-2 was a major haplotype accounting for 71.5% frequency in the natural population, followed by *Hap*-1A-1 with a frequency of 22.3%, and *Hap*-1A-3 with the lowest percentage (6.2%). Association analysis showed that the three haplotypes were significantly associated with PH, PLE, LPN, and TGW (**Table 2**). *Hap*-1A-1 had the lowest PH, PLE, and LPN among the three haplotypes in almost all the environments, with significant difference (*P* < 0.01) between *Hap*-1A-1 and the others. The TGW of *Hap*-1A-1/2 was higher than *Hap*-1A-3 in 10 environments, and the differences were significant in seven environments (*P* < 0.01 or 0.05) (**Figure 4**). Therefore, *Hap*-1A-1 could be a superior allele for increasing TGW and reducing PH.

**Table 2:** *TaSnRK2.3-1A* haplotypes associated with agronomic traits in 10 environments.

Environments	PH		PLE		LPN		TGW	
	*P*-value	*PVE* (%)	*P*-value	*PVE* (%)	*P*-value	*PVE* (%)	*P*-value	*PVE* (%)
2010-CP-WW	2.58E-06^∗∗∗^	13.56	9.11E-06^∗∗∗^	12.17	3.33E-05^∗∗∗^	10.75	n.s.	–
2010-CP-DS	8.77E-07^∗∗∗^	14.76	1.31E-06^∗∗∗^	14.31	0.0048^∗∗^	5.46	0.0343^∗^	3.42
2010-SY-WW	1.29E-05^∗∗∗^	11.83	1.17E-05^∗∗∗^	11.93	1.40E-04^∗∗∗^	9.22	0.0177^∗^	4.10
2010-SY-DS	1.01E-06^∗∗∗^	14.59	2.86E-05^∗∗∗^	10.91	9.85E-07^∗∗∗^	14.62	0.0302^∗^	3.55
2011-SY-WW	4.08E-07^∗∗∗^	15.62	8.92E-06^∗∗∗^	12.19	8.74E-06^∗∗∗^	12.21	0.029^∗^	3.59
2011-SY-DS	8.66E-07^∗∗∗^	14.78	2.93E-04^∗∗∗^	8.41	8.37E-05^∗∗∗^	9.75	0.005^∗∗^	5.41
2012-CP-WW	7.55E-06^∗∗∗^	12.39	0.0037^∗∗^	5.74	0.0017^∗∗^	6.52	n.s.	–
2012-CP-DS	7.13E-06^∗∗∗^	12.46	0.0159^∗^	4.21	0.0017^∗∗^	6.53	n.s.	–
2012-SY-WW	7.71E-07^∗∗∗^	14.94	1.93E-05^∗∗∗^	11.36	5.34E-04^∗∗∗^	7.77	n.s.	–
2012-SY-DS	2.80E-06^∗∗∗^	13.48	0.0278^∗^	3.64	0.0011^∗∗^	7.00	0.0134^∗^	4.39

*PH, plant height; PLE, peduncle length; LPN, length of penultimate node; TGW, 1,000-grain weight; n.s., not significant; ^∗^*P* < 0.05, ^∗∗^*P* < 0.01, and ^∗∗∗^*P* < 0.001; *PVE*, phenotypic variation explained. The environments were at Changping (CP) and Shunyi (SY) under well-watered (WW) and drought-stressed (DS) conditions in 2010–2012*.

### Association Analysis between Haplotypes of *TaSnRK2.3-1B* and Agronomic Traits

**FIGURE 5:**
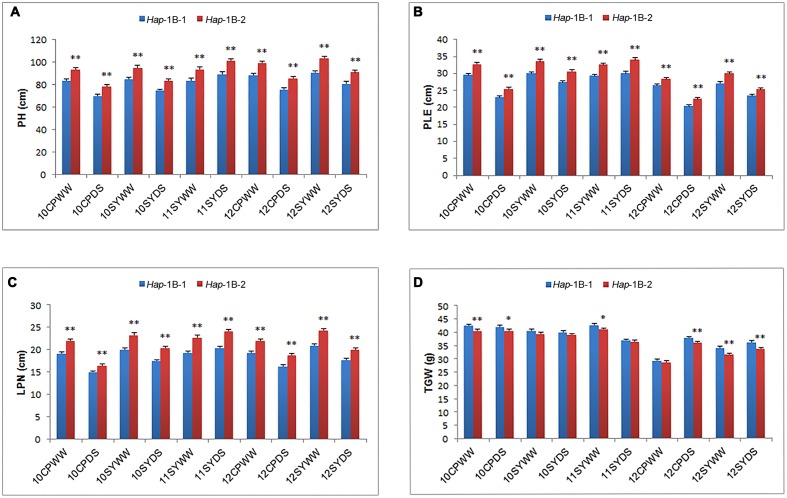
**Phenotypic comparisons of two *TaSnRK2.3-1B* haplotypes in 10 environments**. Traits include PH **(A)**, PLE **(B)**, LPN **(C)**, and TGW **(D)**. ^∗,^^∗∗^ indicate significance at *P* = 0.05 and 0.01, respectively. Error bars denote SE. See footnote to **Table 3** for abbreviations.

For *TaSnRK2.3-1B*, *Hap*-1B-1 and *Hap*-1B-2 were two major haplotypes, accounting for 46.1 and 53.5% frequency. *Hap*-1B-3 was a rare haplotype only presented in one accession, thus it was not included in subsequent statistical analysis. Significant associations were identified between *TaSnRK2.3-1B* haplotypes and agronomic traits, including PH, PLE, and LPN in all 10 environments, and TGW in five environments (**Table 3**). *Hap*-1B-1 was significantly associated with lower PH, PLE, and LPN, and higher TGW (**Figure 5**). Therefore, *Hap*-1B-1 might be a favorable haplotype in terms of PH and TGW.

**Table 3:** *TaSnRK2.3-1B* haplotypes associated with agronomic traits in 10 environments.

Environments	PH		PLE		LPN		TGW	
	*P*-value	*PVE* (%)	*P*-value	*PVE* (%)	*P*-value	*PVE* (%)	*P*-value	*PVE* (%)
2010-CP-WW	4.83E-05^∗∗∗^	17.10	9.39E-04^∗∗∗^	11.21	1.98E-08^∗∗∗^	33.65	0.0201^∗^	5.48
2010-CP-DS	5.32E-05^∗∗∗^	16.90	9.23E-04^∗∗∗^	11.24	1.44E-05^∗∗∗^	19.57	n.s.	–
2010-SY-WW	1.45E-04^∗∗∗^	14.92	0.0013^∗∗^	10.62	1.06E-07^∗∗∗^	30.14	n.s.	–
2010-SY-DS	2.08E-04^∗∗∗^	14.17	0.0025^∗∗^	9.31	9.83E-08^∗∗∗^	30.12	n.s.	–
2011-SY-WW	7.16E-04^∗∗∗^	11.74	0.0023^∗∗^	9.52	1.13E-06^∗∗∗^	24.90	0.0316^∗^	4.67
2011-SY-DS	6.84E-05^∗∗∗^	16.40	1.04E-04^∗∗∗^	15.57	4.98E-07^∗∗∗^	26.65	n.s.	–
2012-CP-WW	7.01E-05^∗∗∗^	16.36	0.0235^∗^	5.20	9.15E-05^∗∗∗^	15.83	n.s.	–
2012-CP-DS	3.58E-05^∗∗∗^	17.73	0.0021^∗∗^	9.67	4.15E-08^∗∗∗^	32.09	0.0171^∗^	5.76
2012-SY-WW	1.36E-05^∗∗∗^	19.73	0.0018^∗∗^	9.97	1.51E-07^∗∗∗^	29.25	0.0039^∗∗^	8.49
2012-SY-DS	2.95E-04^∗∗∗^	13.49	0.0279^∗^	4.89	2.31E-05^∗∗∗^	18.62	7.19E-04^∗∗∗^	11.73

*PH, plant height; PLE, peduncle length; LPN, length of penultimate node; TGW, 1,000-grain weight; n.s., not significant; ^∗^*P* < 0.05, ^∗∗^*P* < 0.01, and ^∗∗∗^*P* < 0.001; *PVE*, phenotypic variation explained. The environments were at Changping (CP) and Shunyi (SY) under well-watered (WW) and drought-stressed (DS) conditions in 2010–2012*.

### Association Analysis between Haplotypes of *TaSnRK2.3-1B* and SWSC

**FIGURE 6:**
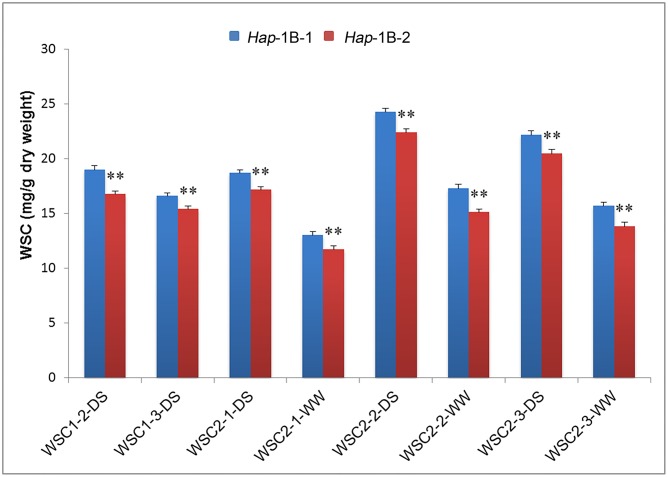
**Comparison of stem water-soluble carbohydrates (SWSC) associated with haplotypes of *TaSnRK2.3-1B* at different grain filling stages**. ^∗∗^ indicates significance at *P* = 0.01. Error bars denote SE. See footnote to **Table 4** for description of traits.

For *TaSnRK2.3-1B*, significant associations were detected between the two haplotypes and SWSC, including WSC1-2-DS, WSC1-3-DS, WSC2-1-DS, WSC2-2-DS/WW, and WSC2-3-DS/WW (**Table 4**). *Hap*-1B-1 had higher SWSC than *Hap*-1B-2 at flowering and mid-grain filling stages with significant difference (*P* < 0.01) (**Figure 6**). So *Hap*-1B-1 might be a potential haplotype to increase SWSC.

**Table 4:** *TaSnRK2.3-1B* haplotypes associated with stem water-soluble carbohydrates (SWSC) at different grain-filling stages.

Traits	*P*-Value	*PVE* (%)	Traits	*P*-Value	*PVE* (%)	Traits	*P*-Value	*PVE* (%)
WSC1-1-DS	n.s.	–	WSC2-1-DS	0.0016^∗∗^	10.25	WSC3-1-DS	n.s.	–
WSC1-2-DS	5.82E-07^∗∗∗^	26.36	WSC2-2-DS	0.0012^∗∗^	10.83	WSC3-2-DS	n.s.	–
WSC1-3-DS	2.65E-04^∗∗∗^	13.69	WSC2-3-DS	0.0013^∗∗^	10.59	WSC3-3-DS	n.s.	–
WSC1-1-WW	n.s.	–	WSC2-1-WW	n.s.	–	WSC3-1-WW	n.s.	–
WSC1-2-WW	n.s.	–	WSC2-2-WW	0.0030^∗∗^	8.97	WSC3-2-WW	n.s.	–
WSC1-3-WW	n.s.	–	WSC2-3-WW	0.0028^∗∗^	9.12	WSC3-3-WW	n.s.	–

*WSC1-1-DS/WW, WSC1-2-DS/WW, and WSC1-3-DS/WW, WSC content of peduncle, lower stem internode and total stem under drought-stressed/well-watered at flowering stage, respectively. WSC2-1-DS/WW, WSC2-2-DS/WW, and WSC2-3-DS/WW, WSC content of peduncle, lower stem internode and total stem under drought-stressed/well-watered at mid-grain filling stage, respectively. WSC3-1-DS/WW, WSC3-2-DS/WW, and WSC3-3-DS/WW indicate WSC content of peduncle, lower stem internode and total stem under drought-stressed/well-watered at maturity stage. n.s., not significant; ^∗∗^*P* < 0.01 and ^∗∗∗^*P* < 0.001; *PVE*, phenotypic variation explained*.

### Geographic Distribution of Haplotypes of *TaSnRK2.3-1A* and *TaSnRK2.3-1B* in 10 Chinese Wheat Production Zones

**FIGURE 7:**
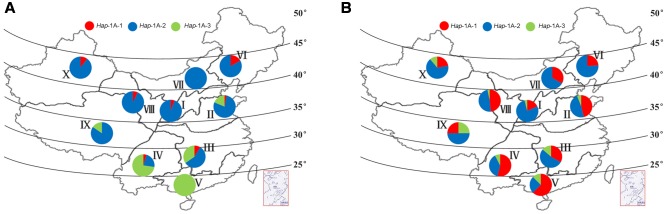
**Haplotype distribution of *TaSnRK2.3-1A* in 10 Chinese wheat ecological regions. (A)** Distribution of *TaSnRK2.3-1A* haplotypes in 157 Chinese landraces. **(B)** Distribution of *TaSnRK2.3-1A* haplotypes in 348 modern cultivars. I, Northern winter wheat region; II, Yellow and Huai River valley winter wheat region; III, Low and middle Yangtze River valley winter wheat region; IV, Southwestern winter wheat region; V, Southern winter wheat region; VI, Northeastern spring wheat region; VII, Northern spring wheat region; VIII, Northwestern spring wheat region; IX, Qinghai-Tibet spring-winter wheat region; X, Xinjiang winter-spring wheat region.

**FIGURE 8:**
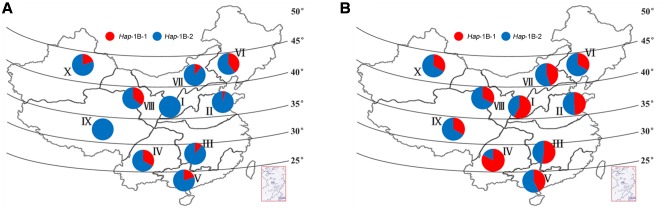
**Distribution of *TaSnRK2.3-1B* haplotypes in 10 Chinese wheat ecological regions. (A)** Distribution of *TaSnRK2.3-1B* haplotypes in 157 Chinese landraces. **(B)** Distribution of *TaSnRK2.3-1B* haplotypes in 348 modern cultivars. See footnote to **Figure 7** for abbreviations from I to X.

The Chinese wheat production area is classified into 10 main agro-ecological zones based on cultivar ecotypes, growing season, and cultivar response to temperature and photoperiod (Zhang et al., 2002). Among landraces, selection pressure on haplotypes in the different zones was not as strong as expected, and the frequencies of the favored haplotype *Hap*-1A-1 was generally low, none was identified in zone V, VII, and IX (**Figure 7A**). Similar trends were also observed for *TaSnRK2.3-1B* (**Figure 8A**).

AS shown in **Figures 7**, **8**, from Chinese landraces to modern cultivars, the frequencies of the two superior haplotypes *Hap*-1A-1 and *Hap*-1B-1 increased across almost all zones except in VI (40% to 33%) and VIII (36% to 33%) of *Hap*-1B-1. *Hap*-1B-1 was the most favorable haplotype in major wheat production regions I (58%), II (49%), III (52%), and IV (83%) compared to the other six regions in Population 3. The results indicated the two haplotypes had suffered strong positive selection in Chinese wheat breeding programs.

### *Hap*-1A-1 and *Hap*-1B-1 Were Positively Selected in the Process of Chinese Wheat Breeding

**FIGURE 9:**
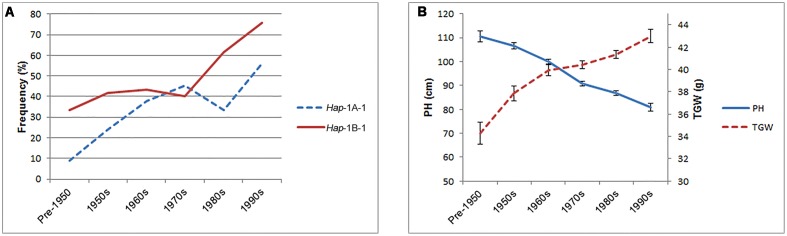
**Elite haplotypes *Hap*-1A-1 and *Hap*-1B-1 were positively selected in Chinese wheat breeding programs. (A)** The frequencies of two favored haplotype in Population 3 over decades. 12, 25, 55, 102, 106, and 34 accessions were released in the pre-1950, 1950s, 1960s, 1970s, 1980s, and 1990s, respectively. Fourteen accessions with unknown release dates were excluded. **(B)** The changes of PH and TGW in Population 3 over decades. Error bars denote 2 × SE.

Three hundred and forty-eight Chinese modern cultivars were divided into six subgroups according to 10-year release intervals to evaluate changes in haplotype frequencies over time. As a whole, the proportions of the two favored haplotypes, *Hap*-1A-1 and *Hap*-1B-1, increased gradually with PH reduction and TGW enhancement in Chinese modern cultivars since pre-1950 (**Figure 9**), suggesting that these favored haplotypes experienced positive selection in the process of wheat breeding. Furthermore, *Hap*-1B-1 (up to 75.9%) was positively selected by wheat breeders, while *Hap*-1A-1 (only 55.9%) still has considerable potential for further utilization.

## Discussion

In this study, we obtained the three genomic sequences of *TaSnRK2.3-1A*, *TaSnRK2.3-1B*, and *TaSnRK2.3-1D*; however, there was no polymorphism identified in *TaSnRK2.3-1D*, therefore, *TaSnRK2.3-1D* was not included for polymorphism analysis. DNA polymorphism analysis results revealed that the variations for *TaSnRK2.3-1A* only occurred in non-exon regions, while the variations for *TaSnRK2.3-1B* occurred in both introns and exons. Further association analysis indicated both *TaSnRK2.3-1A* and *TaSnRK2.3-1B* were significantly associated with PH, PLE, LPN, and TGW, thus we speculate that the two genes might have similar functions. Further studies need to be performed to validate the allelic effects of these polymorphisms, such as gene expression analysis.

Stem water-soluble carbohydrates are not only a main source for grain filling, but also crucial osmolytes in regulating cell turgor under abiotic stress conditions in wheat (Yang et al., 2007). Association analysis results showed that *TaSnRK2.3-1B* was associated with SWSC under three WW conditions and five DS conditions, which was similar to previous studies of *TaSnRK2.7-B* and *TaSnRK2.8-A* in SWSC metabolism (Zhang et al., 2011a,b, 2013). Since *TaSnRK2.3*, *TaSnRK2.7*, and *TaSnRK2.8* belong to SnRK2 subfamily, they might function similarly in carbohydrate metabolism. Moreover, SWSC under DS conditions were clearly higher than SWSC under corresponding WW conditions, which agrees with the observation that drought induced WSC remobilization increases in response to water deficit (Goggin and Setter, 2004; Rebetzke et al., 2008).

Compelling evidence demonstrates that yeast SNF1-kinase and mammalian AMPK, and plant SnRK1 participate in sugar metabolism, including starch synthesis and carbohydrate distribution (Halford et al., 2003; Schwachtje et al., 2006). Further data support that SnRK2 and SnRK3 originated by duplication of SnRK1 and then diverged rapidly during plants response to adverse stresses (Hrabak et al., 2003; Hauser et al., 2011). However, our results support that *TaSnRK2.3* is involved in both abiotic stress responses and SWSC metabolism, suggesting it still maintains ancient functions, which was also observed in *TaSnRK2.7* and *TaSnRK2.8* (Zhang et al., 2011a).

For *TaSnRK2.3-1B*, the favored haplotype *Hap*-1B-1 had the higher TGW and higher SWSC at flowering and mid-grain filling stages, consistent with earlier results that there were significant correlations between TGW and SWSC (Zhang et al., 2014; Li et al., 2015). Li et al. (2015) indicated that SWSC can make a positive contribution to TGW under variable water conditions. High SWSC were suggested as an useful trait for improving grain weight in wheat breeding (Shearman et al., 2005; Ruuska et al., 2006). High grain yield is one of the most important breeding objectives in wheat improvement. The high heritability values of TGW have proved it is phenotypically the most stable yield component which continuously attracts the attention of breeders.

In our previous study, *TaSnRK2.3-1B* was mapped to a region flanked by *wmc156* (2.1 cM) and *p3446-183* (2.9 cM) on chromosome 1B in the DH population derived from a cross of Hanxuan 10 × Lumai 14, co-located with a quantitative trait locus controlling PH (Wu et al., 2010, 2012; Tian et al., 2013). Here, our association data demonstrated that *TaSnRK2.3-1B* was significantly associated with PH, and the favored haplotypes had lower PH. Additionally, as shown in **Figure 9B**, PH decreased in stepwise manner over decades and was positively selected in the process of Chinese wheat breeding. Therefore, we speculate that *TaSnRK2.3-1B* might be a potential gene related to PH or closely linked with genes involved in PH regulation.

Among various markers, functional markers derived from polymorphic sites within target genes, are superior to conventional molecular markers such as RFLPs, SSRs, and AFLPs because of complete linkage with trait locus alleles, and are ideal for marker-assisted breeding. In the current study, functional markers were developed for genotyping based on variants in *TaSnRK2.3* genes. In sum, the CAPS/dCAPS markers 2.3AM1, 2.3AM2, 2.3BM1, and 2.3BM2 were used successfully. Combinations of the two markers 2.3AM1 and 2.3AM2 for A genome formed three haplotypes that significantly affected important agronomic traits, similarly to other two markers 2.3BM1 and 2.3BM2 in B genome. These four markers are all co-dominant and allow efficient assays of large DNA samples in a simple, rapid, and low-cost procedure, which is performed in most molecular biology and/or plant breeding laboratories.

To sum up, the two elite haplotypes *Hap*-1A-1 and *Hap*-1B-1 of *TaSnRK2.3s*, can contribute positively to grain size enhancement and PH reduction in wheat. They could be applied in wheat breeding programs using marker assisted selection.

## Author Contributions

Conceived the idea: RJ, XM, LM, and ZW. Performed the experiments: LM, XM, JW, ZL, BZ, WL, and XC. Analyzed the data: LM, XM, and RJ. Wrote the manuscript: LM and XM. Revised the manuscript: RJ, XM, and MR.

## Conflict of Interest Statement

The authors declare that the research was conducted in the absence of any commercial or financial relationships that could be construed as a potential conflict of interest.

## Footnotes

1 http://helix.wustl.edu/dcaps/dcaps.html
